# Identification of novel PHD-finger genes in pepper by genomic re-annotation and comparative analyses

**DOI:** 10.1186/s12870-022-03580-2

**Published:** 2022-04-20

**Authors:** Ji-Yoon Guk, Min-Jeong Jang, Seungill Kim

**Affiliations:** Department of Environmental Horticulture, University of Seoul, Seoul, 02504 Republic of Korea

**Keywords:** PHD-finger, Re-annotation, Gene family, Pepper, Abiotic stress

## Abstract

**Background:**

The plant homeodomain (PHD)-finger gene family that belongs to zinc-finger genes, plays an important role in epigenetics by regulating gene expression in eukaryotes. However, inaccurate annotation of PHD-finger genes hinders further downstream comparative, evolutionary, and functional studies.

**Results:**

We performed genome-wide re-annotation in *Arabidopsis thaliana* (*Arabidopsis*), *Oryza sativa* (rice), *Capsicum annuum* (pepper), *Solanum tuberosum* (potato), and *Solanum lycopersicum* (tomato) to better understand the role of PHD-finger genes in these species. Our investigation identified 875 PHD-finger genes, of which 225 (26% of total) were newly identified, including 57 (54%) novel PHD-finger genes in pepper. The PHD-finger genes of the five plant species have various integrated domains that may be responsible for the diversification of structures and functions of these genes. Evolutionary analyses suggest that PHD-finger genes were expanded recently by lineage-specific duplication, especially in pepper and potato, resulting in diverse repertoires of PHD-finger genes among the species. We validated the expression of six newly identified PHD-finger genes in pepper with qRT-PCR. Transcriptome analyses suggest potential functions of PHD-finger genes in response to various abiotic stresses in pepper.

**Conclusions:**

Our data, including the updated annotation of PHD-finger genes, provide useful information for further evolutionary and functional analyses to better understand the roles of the PHD-finger gene family in pepper.

**Supplementary Information:**

The online version contains supplementary material available at 10.1186/s12870-022-03580-2.

## Background

Structural annotation of protein-coding genes is a fundamental process for obtaining essential genetic information for further evolutionary and functional analyses [1]. However, previous annotations omitted numerous protein-coding genes, interfering with accurate downstream analyses [2, 3]. Specifically, protein-coding gene omission is frequently observed for gene families that exist in high copy numbers and specific species in genomes [4, 5]. To update annotations containing those missing protein-coding genes, previous studies have performed re-annotation of protein-coding genes in plant and animal genomes using recently developed annotation tools [6–10]. The results demonstrate the importance of continuous updates to the annotations, as many protein-coding genes involved in the biological characteristics of a species.

The plant homeodomain (PHD)-finger proteins are widely distributed in eukaryotes [11], with most PHD-finger proteins found in the nucleus [12]. PHD-finger proteins possess one or more PHD-finger domains, which comprise approximately 60 amino acids consisting of the conserved Cys4-His-Cys3 zinc-binding motif [11, 13–15] that is stabilized by binding to two zinc ions [16]. Since discovery of the first PHD-finger protein, HAT3.1, in *Arabidopsis* [17], many studies have revealed that PHD-finger proteins function as epigenetic readers that recognize and bind to histones with unmodified or post-translational modifications (PTMs), transform chromatin structure, and regulate the activation or repression of gene transcription [18–24]. In addition, PHD-finger genes are known to be involved in reproductive and developmental processes. In *Arabidopsis*, the MALE STERILITY1 (MS1) and DUET proteins participate in reproduction by regulating the transcription of genes associated with male gametogenesis and male meiosis, respectively [25, 26]. PICKLE (PKL) is involved in repressing embryonic trait gene expression during development by remodeling chromatin structure [27]. PKL also plays an important role in response to cold and salt stress [28, 29]. In rice, Early heading date 3 (Ehd3) and HAZ1 act as transcription factors involved in the regulation of flowering and gibberellin (GA) signaling, respectively [30, 31]. However, the roles of the PHD-finger gene family have yet to be studied in several important agricultural crops.

In this study, we conducted re-annotation and comparative analyses of PHD-finger genes in five plant genomes: *Arabidopsis thaliana* (*Arabidopsis*), *Oryza sativa* (rice), *Capsicum annuum* (pepper), *Solanum tuberosum* (potato), and *Solanum lycopersicum* (tomato). We identified 875 PHD-finger genes, including 225 genes (26%) that were missed in previous annotations. Domain architecture analysis revealed that integration of diverse domains could contribute to the structural and functional diversification of PHD-finger genes. Based on phylogenetic analysis, PHD-finger genes were classified into 14 subgroups with distinct domain architectures (G1 ~ G14). Duplication history analysis revealed that most of the potato and pepper PHD-finger genes were expanded recently via lineage-specific duplication. Microsynteny analysis in the Solanaceae species revealed that most of the G6 genes of potato on chromosome 1 were expanded by recent tandem duplication, resulting in diverse copy number variations in Solanaceae species. We validated the expression of newly identified pepper PHD-finger genes by qRT-PCR. Expression clustering analysis and gene ontology (GO) enrichment testing revealed that pepper PHD-finger genes might be associated with binding or regulation-related functions in response to abiotic stresses. Our study demonstrates a comprehensive evolutionary relationship of the PHD-finger gene family between pepper and the other four plant genomes, thus providing fundamental genomic resources that can be used to accelerate further functional agricultural research.

## Results and discussion

### Re-annotation of PHD-finger gene family in pepper and other species

To update and construct a more accurate annotation of PHD-finger genes, we performed a re-annotation and obtained a total of 875 PHD-finger genes in five plant genomes. Of them, 225 genes (26%) were newly identified. Specifically, 57 (54%) pepper PHD-finger genes were newly annotated, indicating that the re-annotation process could improve previous annotations of PHD-finger genes via new gene identification, especially in the pepper genome (Table 1). Many previous studies have addressed the importance of updating numerous omitted genes via re-annotation [6–10]. In this study, we updated more accurate annotations of protein-coding genes by using the novel gene annotation platform for re-annotation, and downstream analysis was performed based on the updated annotations. The number of PHD-finger genes in *Arabidopsis*, rice, and potato was approximately twice those in pepper and tomato (Table 1). The length of PHD-finger proteins varied from 52 to 2724 amino acids, with an average of 541 amino acids, implying that PHD-finger genes encoded proteins with diverse structures (Table 1 and Table S2).

**Table 1:** The number of re-annotated PHD-finger genes in the five plants

Species	Previously annotated genes	Newly annotated genes	Total
*Arabidopsis*	241 (553 aa)	16 (387 aa)	257 (542 aa)
Rice	147 (556 aa)	64 (363 aa)	211 (498 aa)
Pepper	49 (890 aa)	57 (507 aa)	106 (684 aa)
Potato	160 (371 aa)	49 (517 aa)	209 (405 aa)
Tomato	53 (844 aa)	39 (687 aa)	92 (777 aa)
Total	650 (558 aa)	225 (491 aa)	875 (541 aa)

**Fig. 1:**
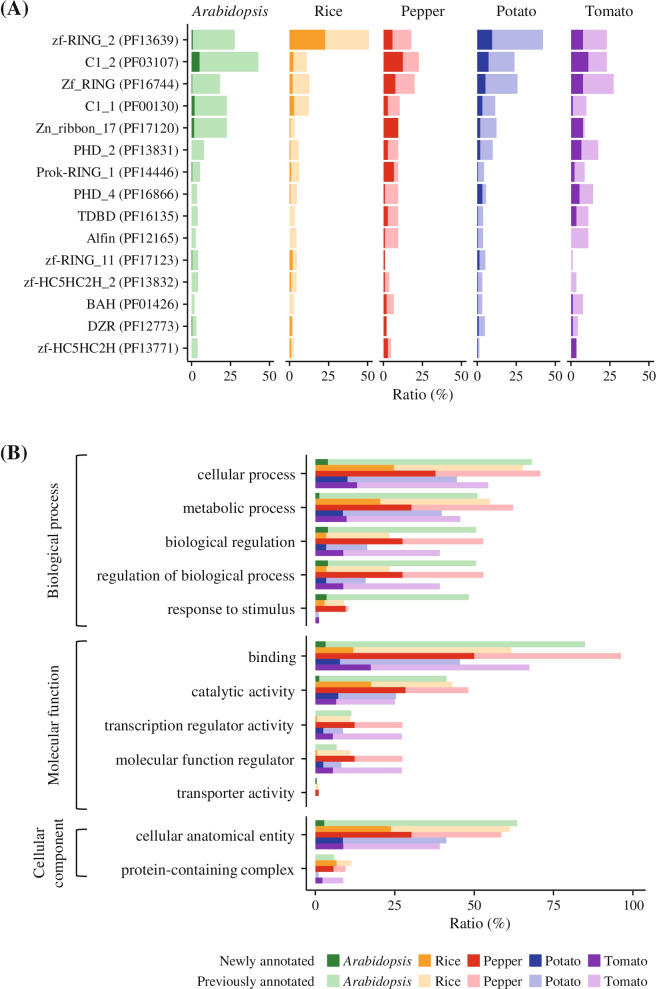
Characterization of the PHD-finger gene family in five plant species. **A**, **B** The proportion of PHD-finger genes for each species is shown in different colors. The proportion of newly identified PHD-finger genes is shown in opaque colors. **A** Integrated domain repertoires of PHD-finger genes. The portion of PHD-finger genes that contained the top 15 integrated domains (IDs) is shown in the bar plot. **B** Distribution of gene ontology (GO) terms of PHD-finger genes. The three main GO categories are listed on the left side of the bar plot. The top five GO terms in each category are shown in bar plot

We then analyzed the domain architecture of PHD-finger genes (Fig. 1). In total, 98% of PHD-finger genes had diverse integrated domains (IDs) such as zf-RING_2 (PF13639), C1_2 (PF03107), and Zf_RING (PF16744) (Fig. 1A and Table S3). When we compared the proportion of IDs within the five species, PHD-finger genes shared a similar predominant ID repertoire; however, the detailed proportion of IDs in each species was distributed unevenly (Fig. 1A). In *Arabidopsis*, rice, and potato, which possess relatively more PHD-finger genes than other species, most of the PHD-finger genes contained specific IDs, such as C1_2 (PF03107) and zf-RING_2 (PF13639). In particular, more than half the rice PHD-finger genes (51%) had zf_RING_2 (PF13639) (Fig. 1A). Notably, most IDs in newly annotated pepper PHD-finger genes consisted of C1_2 (PF03107) and Zf_RING (PF16744). In particular, Zn_ribbon_17 (PF17120) was present only in newly annotated pepper PHD-finger genes (Fig. 1A). These results suggest that diverse IDs could contribute to the structural and functional diversification of the PHD-finger gene family in these five plant species.

Functional annotation based on GO analysis was performed to characterize the putative function of PHD-finger genes in the five plant genomes. We determined GO terms for 760 (87%) PHD-finger genes and categorized them based on molecular function, biological process, and cellular component (Fig. 1B). The predominant terms for molecular function, biological process, and cellular component were ‘binding’ (607; 80%), ‘cellular process’ (531; 70%), and ‘cellular anatomical entity’ (476; 63%), respectively (Fig. 1B). Most of the pepper PHD-finger genes (96%), including newly identified pepper PHD-finger genes (93%), belonged to the ‘binding’ group. These findings were consistent with previously reported functions of PHD-finger genes. For example, the *Arabidopsis* PHD-finger proteins SHL and EBS have been shown to participate in the repression of flowering by recognizing a specific epigenetic mark (H3K4me2/3) in chromatin and binding to floral integrators, SUPPRESSOR OF OVEREXPRESSION OF CO1 (SOC1) and FLOWERING LOCUS T (FT) [32, 33]. Our results suggest that most of the newly identified pepper PHD-finger genes may also be involved in a binding function. Besides these GO terms, PHD-finger genes were annotated to various GO terms, such as metabolic process, catalytic activity, biological regulation, indicating that PHD-finger genes might be implicated in diverse functions. Taken together, our analyses demonstrate that updating the annotation of PHD-finger genes could provide more comprehensive information for more accurate downstream analyses, especially in pepper.

### Phylogenetic analysis of PHD-finger genes in pepper and other species

**Fig. 2:**
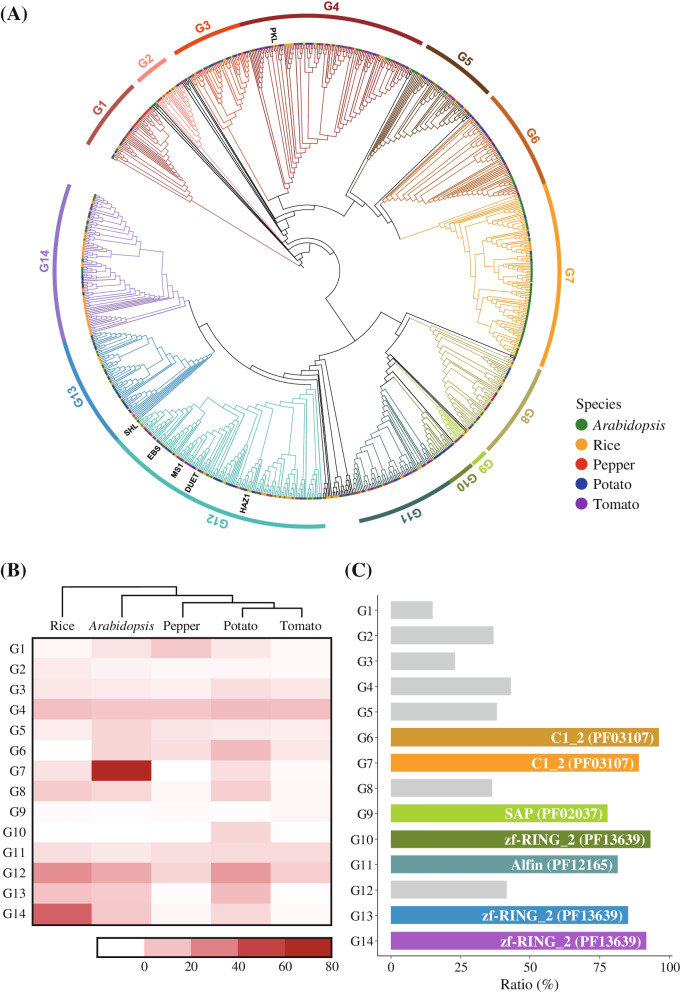
Phylogenetic relationship of PHD-finger genes and characteristics of the 14 subgroups. **A** The phylogenetic tree of PHD-finger genes in the five plant species is depicted. The colored bars outside of the tree represent divided subgroups. Different colors at branch tips indicate different species. Known functional genes are labeled on the outer edge. **B** The numbers of PHD-finger genes in each subgroup are shown in a heatmap. **C** Major integrated domains in subgroups. Colored bars indicate groups with more than 75% integrated domains. Each bar is colored with the same colors of subgroups in phylogenetic tree. Pfam IDs of the main integrated domain are labeled in the bar plot

To explore the evolutionary relationships of PHD-finger genes in the five plant species, we constructed a phylogenetic tree using the re-annotated PHD-finger genes (Fig. 2A). Based on the phylogeny and domain architectures, the PHD-finger gene family was classified into 14 subgroups (Fig. 2A). Most of the *Arabidopsis* and rice PHD-finger genes were specifically clustered in G7 and G14, respectively (Fig. 2B). We observed many of pepper PHD-finger genes of G1 and most of them were newly identified pepper PHD-finger genes, indicating that PHD-finger genes in G1 were expanded in pepper (Fig. 2B). To date, only a few PHD-finger genes were identified in previous functional studies in plants. Functional PHD-finger genes in *Arabidopsis* and rice are known to be involved in the developmental process [25–27, 30, 31]. As shown in Fig. 2A, all except one (PKL) clustered in the same subgroup (G12) even though the PHD-finger genes diverged from various lineages (Fig. 2A). Considering the phylogenetic tree, our findings suggest that the re-annotated PHD-finger genes derived from different lineages could be novel resources for exploring the distinct roles of PHD-finger genes across various plant species.

Furthermore, we found that PHD-finger genes clustered in the same subgroup exhibited similar domain architectures, sharing a major integrated domain (ID). This suggests that the majority of PHD-finger genes in the same subgroup had expanded after domain integration. We observed specific IDs that consisted mainly of seven subgroups (G6, G7, G9, G10, G11, G13, and G14) (Fig. 2C and Table S3). The PHD-finger genes with zf_RING_2 (PF13639) were most abundant, found in 93%, 85%, and 92% of the total PHD-finger genes in G10, G13, and G14, respectively (Fig. 1A and 2C). The PHD-finger genes with the second most ID, C1_2 (PF03107), were clustered in G6 and G7 (Fig. 1A and 2C). In addition, SAP (PF02037) and Alfin (PF12165) were observed in most of PHD-finger genes belonging to G9 and G11, respectively (Fig. 2C). These results suggest that PHD-finger genes having specific IDs were lineage-specifically expanded and preserved in specific subgroups.

### Duplication history of PHD-finger genes

**Fig. 3:**
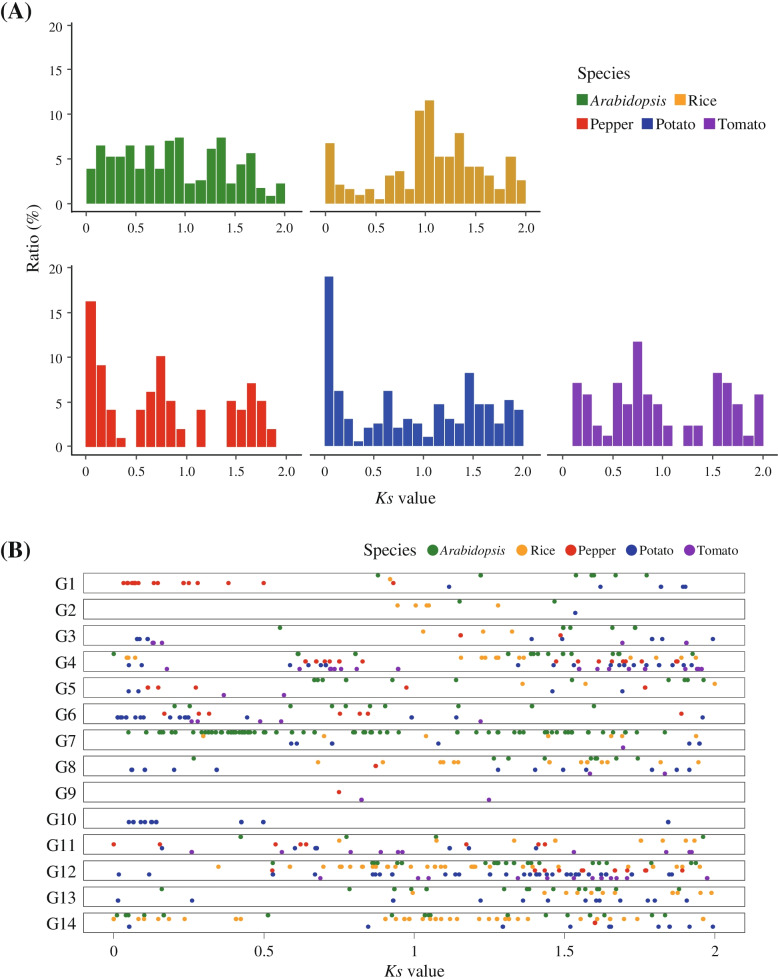
Distribution of *Ks* values between duplicated PHD-finger gene pairs in the five plant species. **A, B** The different colors represent different species. **A** The frequency proportions of *Ks* values in each species are shown as a bar plot. **B** The distribution of *Ks* values in each subgroup is presented in a dot plot

Gene duplication is one key mechanism that contributes to the diversification of gene repertoires through the expansion of the copy number of genes [34]. To infer the duplication period of PHD-finger genes in five plants, we estimated the gene duplication time based on *Ks* values between duplicated gene pairs in each subgroup (Fig. 3A). Distinctly, the *Ks* values of many PHD-finger genes in potato were less than 0.1, indicating that these genes emerged by recent gene duplication after speciation with tomato (Fig. 3A) [35]. Despite the relatively low number of PHD-finger genes in pepper, a high proportion of these genes also underwent gene duplication recently (Fig. 3A). These results suggest that those recently duplicated PHD-finger genes in potato and pepper are species-specific and contributed to the diversification of PHD-finger gene repertoires in each species. We further investigated the distribution of *Ks* values of the duplicated PHD-finger genes in 14 subgroups (Fig. 3B). Most of the recently duplicated PHD-finger genes in potato and pepper were clustered in specific subgroups (Fig. 3B). In pepper, these genes were newly identified from the re-annotation analysis conducted in this study and were mainly clustered in the G1 subgroup (Fig. 3B). In potato, most of the recently duplicated PHD-finger genes were clustered in G6 and G10 (Fig. 3B). These results indicate that a large proportion of potato and pepper PHD-finger genes in specific subgroups recently emerged by lineage-specific duplication, leading to expansion of the PHD-finger gene family, especially in potato.

**Fig. 4:**
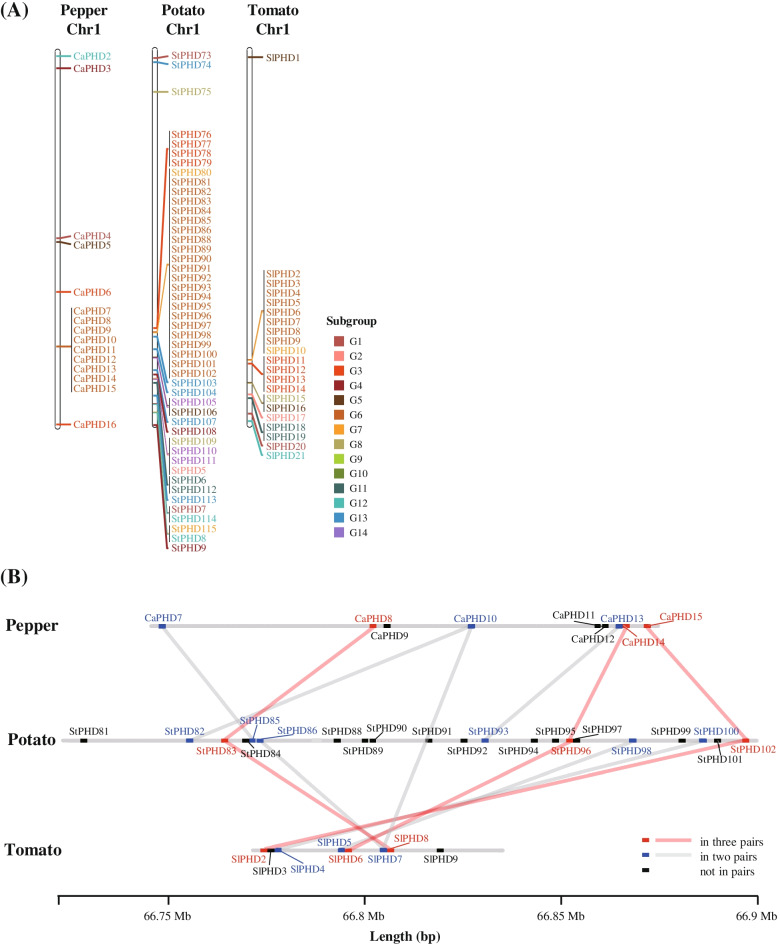
Location and synteny of PHD-finger genes on chromosome 1 of pepper, potato, and tomato. **A** Distribution of the PHD-finger genes on chromosome 1 of pepper, potato, and tomato. PHD-finger genes of G6 were clustered in specific regions of chromosome 1 of pepper (9 genes), potato (21 genes), and tomato (8 genes). Gene names are written in the same colors of subgroups in the phylogenetic tree. **B** Synteny analysis of G6 genes in chromosome 1. Orthologous genes in three species are labeled in red and connected by red lines. Orthologous genes in two species are marked in blue and connected by gray lines while others are shown in black

When we investigated the chromosomal location of PHD-finger genes, we found that, except for genes in specific subgroups, most were evenly distributed throughout the chromosomes. Pepper PHD-finger genes in G1, which had recently expanded, were located on chromosomes 1, 2, 3, 4, 6, 7, and 12 (Fig. S1). Several of the potato PHD-finger genes were positioned on chromosome 1 where they formed a tandem array in the long arm, but most were contained in G6 (Fig. 4A). We also observed that the PHD-finger genes in G6 of pepper and tomato were clustered in the corresponding regions of chromosome 1 as PHD-finger genes in potato (Fig. 4A). In these regions, the PHD-finger genes were detected in the different number of gene copies in pepper (9), potato (21), and tomato (8), indicating that copy number variations of PHD-finger genes of G6 located on chromosome 1 occurred in these species (Fig. 4A). We further investigated the syntenic genes in these regions and identified three pairs of putative orthologous genes, all preserved in chromosome 1 of all three Solanaceae species during evolution (Fig. 4B). Of the PHD-finger genes in the syntenic region, several genes in pepper (3), potato (12), and tomato (2) had no orthologous genes among the three genomes, indicating that a large number of potato-specific PHD-finger genes were clustered in the syntenic region. Altogether, our results from microsynteny analysis combined with duplication time demonstrate that the PHD-finger genes belonging to G6 were derived from expansion via recent tandem duplication in the potato genome, leading to a diversity in copy number variations in the Solanaceae species.

### Expression analyses of PHD-finger genes in pepper under abiotic stress

**Fig. 5:**
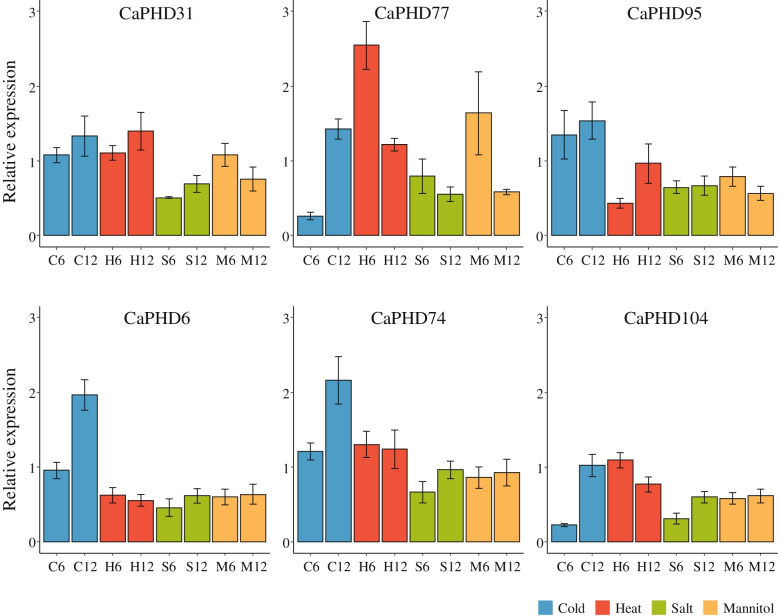
Validation of expression of newly identified pepper PHD-finger genes using qRT-PCR. Each abiotic stress is marked with a different color. The x-axis represents the number of hours (6 h or 12 h) of each abiotic treatment. C: Cold, H: Heat, S: Salt, M: Mannitol, 6: 6 h, 12: 12 h. The error bars indicate the standard error

We first validated the expression of six of the newly identified pepper PHD-finger genes by performing quantitative real-time PCR (qRT-PCR). Our data revealed expression of those genes under abiotic stress treatment after 6 and 12 h (Fig. 5), indicating that these genes are truly expressed under abiotic stress conditions. We then conducted RNA-Seq analysis to investigate the putative function of pepper PHD-finger genes in response to abiotic stress conditions. We estimated expression profiles of PHD-finger genes in pepper using RNA-Seq under cold, heat, salt, and mannitol stresses (Fig. S2). Overall, the pepper PHD-finger genes in G11 and G12 were highly expressed under abiotic stress (Fig. S2) while most of the PHD-finger genes in G6 were expressed at low levels (Fig. S2). Pepper PHD-finger genes in G1 also expressed at lower levels in all abiotic stresses except CaPHD94 (Fig. S2).

**Fig. 6:**
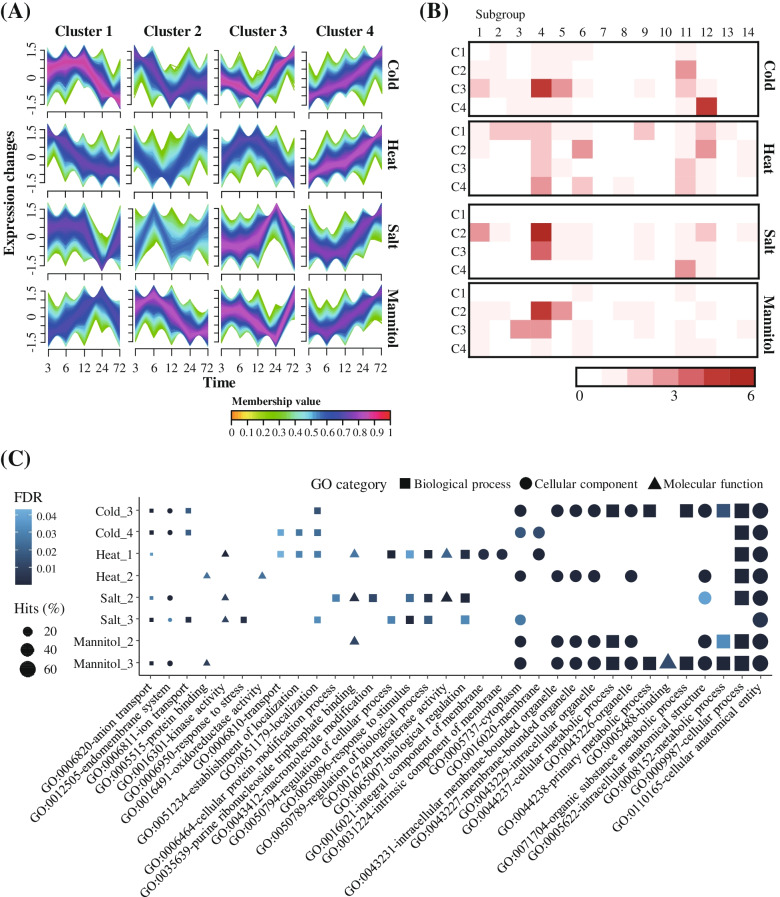
Expression pattern and potential function of differentially expressed genes (DEGs) in pepper treated with different abiotic stresses. **A** Expression patterns of whole pepper DEGs (including PHD-finger DEGs) under abiotic stress conditions is presented as four clusters of each abiotic stress. **B** The number of PHD-finger DEGs in each subgroup is shown in a heatmap. **C** Top 10 GO terms in each major cluster are plotted. The shapes indicate the three main GO categories while the shape size indicates the frequency of the GO terms. FDR, false discovery rate

Next, we then identified differentially expressed genes in pepper, including the newly identified PHD-finger genes, in response to abiotic stresses such as cold (14,698), heat (14,217), salt (12,549), and mannitol (12,513). Our analysis identified 43, 47, 32, and 34 PHD-finger differentially expressed genes (DEGs) in pepper in response to cold, heat, salt, and mannitol treatment, respectively. We conducted expression clustering analysis and grouped these DEGs into four clusters based on their expression pattern under abiotic stress (Fig. 6A). A large proportion of the PHD-finger DEGs were found in G4, and these genes were enriched in a specific cluster for each stress, such as cold cluster 3 (5; 11.6%), heat cluster 4 (3; 6.4%), salt cluster 2 (6; 18.8%), and mannitol cluster 2 (5; 14.7%) (Fig. 6B). These results indicate that, in response to abiotic stress, many PHD-finger DEGs in G4 could participate with other pepper DEGs in specific clusters.

We also performed GO enrichment test of clusters that contained an abundant number of PHD-finger genes (Fig. 6C). Our analyses showed that the pepper DEGs are associated with diverse functions, including cellular anatomical entity (GO:0110165), cellular process (GO:0009987), and metabolic process (GO:0008152) (Fig. 6C). This suggests that these pepper PHD-finger genes could play a variety of roles in response to various abiotic stress conditions. Specifically, binding- or regulation-related GO terms were abundant in some clusters (Fig. 6C). Mannitol cluster 3 included many pepper DEGs related to binding function (GO:0005488) (Fig. 6C). Binding-related GO terms, such as protein binding (GO:0005515) and purine ribonucleoside triphosphate binding (GO:0035639), were also found under heat and salt stress (Fig. 6C). These results suggest that many pepper PHD-finger genes could be involved in regulation of stress-related gene expression by binding to histone modifications under abiotic stress conditions, consistent with a previously known function of PHD-finger genes [28]. Moreover, regulation-related GO terms such as biological regulation (GO:0065007), regulation of biological process (GO:0050789), and regulation of cellular process (GO:0050794) were concentrated in heat cluster 1, salt cluster 2, and salt cluster3 (Fig. 6C). In particular, most of the PHD-finger genes in salt cluster 2 were contained in G4, a subgroup containing *Arabidopsis* PKL (Fig. 2A). A previous study showed that *Arabidopsis pkl* mutants were sensitive to salt stress, decreasing cotyledon greening and root elongation [28]. This suggests that the PHD-finger genes in salt cluster 2 could be involved in regulation of response mechanisms of pepper when exposed to salt stress. In addition, a previous study suggested that *Arabidopsis* PKL regulates the expression of cold-responsive (COR) genes under cold stress [28, 29]. Taken together, our results suggest that the pepper PHD-finger genes could be involved in diverse response mechanisms to various abiotic stresses by interacting with other pepper genes.

## Conclusions

High-quality annotation of protein-coding genes is extremely important and serves as a foundation for comparative analyses of gene families [2, 3]. Because previous annotations contained many of omitted protein-coding genes, a re-annotation process is essential for enabling accurate downstream analysis [4, 5]. In this study, we conducted re-annotation and comparative analyses of PHD-finger gene family in five plant species. Our study provides an improved annotation of PHD-finger genes in these plant genomes, including the identification of 225 (26% of total) novel PHD-finger genes. Notably, over half (54%) of PHD-finger genes in pepper were newly identified in this study, indicating that the re-annotation process could facilitate the discovery of new gene models missing in previous annotations.

In general, evolutionarily conserved domains in protein-coding genes are considered to be significantly related to gene function [36]. When we investigated the domain architecture of re-annotated PHD-finger genes, we found that various structures and functions could be inferred in the PHD-finger genes as a result of integrating diverse domains. Based on the phylogenetic analysis, PHD-finger genes in the five species were clustered into 14 subgroups with distinct domain architectures, indicating that the PHD-finger gene family have diverged from various lineages and expanded lineage specifically with specific integrated domains. Estimation of the duplication time in duplicated PHD-finger gene pairs suggests that recently duplicated PHD-finger genes in potato and pepper were expanded lineage-specifically in specific subgroups. Solanaceae PHD-finger genes in syntenic regions of chromosome 1 have been derived from recent tandem duplication, leading to diverse gene repertoires in the PHD-finger gene family of the Solanaceae species. Our findings could serve as a novel resource for investigating new functions of PHD-finger genes, especially in Solanaceae plants, for which functional studies have yet to be conducted.

We verified via qRT-PCR that newly annotated PHD-finger genes are expressed. Transcriptome analyses and GO enrichment test suggest that many pepper PHD-finger DEGs could participate in binding- or regulation-related functions in response to heat, salt, or mannitol stress.

Taken together, we provide: i) updated genomic resources, containing previously omitted PHD-finger genes in five plant genomes including pepper and ii) a more comprehensive understanding of the structure and function of pepper PHD-finger genes.

## Materials and methods

### Re-annotation of PHD-finger gene family in five plant genomes

We obtained the genome sequences of *Arabidopsis thaliana* [37], *Oryza sativa* [38], *Capsicum annuum* [39], *Solanum tuberosum* [40], and *Solanum lycopersicum* [41], including genome assemblies and annotations (Table S1). Then, we performed a re-annotation analysis of PHD-finger genes using TGFam-Finder v1.20 [8]. The downloaded genome assemblies and protein sequences were used as ‘TARGET_GENOME’ and ‘PROTEIN_FOR_DOMAIN_IDENTIFICATION’, respectively. TSV files containing functional domain information were generated using InterProScan 5 [42] and used as ‘TSV_FOR_DOMAIN_IDENTIFICATION’. The target domain ID of PHD-finger domain was ‘PF00628’ according to the Pfam database (http://pfam.xfam.org/).

We assigned new gene names for re-annotated PHD-finger genes instead of locus tag names in the published annotations that we used. If PHD-finger genes were already given a gene name, we used the same published name [43, 44]. We designated new names for the other genes based on the order in which they appear on the chromosome.

### Identification of integrated domains in PHD-finger genes

To identify integrated domains (IDs) of PHD-finger genes, we used TSV files generated by InterProScan 5 [42] according to the Pfam database (http://pfam.xfam.org/). Domains, except for the PHD-finger domain (PF00628), were considered as integrated domains. The bar plots in Fig. 1A were visualized using ggplot2 [45] in the R software.

### Functional annotation using GO analysis

To predict the putative function of PHD-finger genes, GO annotation was performed using OmicsBox (version 1.4, https://www.biobam.com/omicsbox/). The PHD-finger protein sequences were aligned to the NCBI non-redundant proteins database (nr v5) using BLASTP with an e-value cutoff (< 10^–3^). BLAST results were mapped to and annotated with GO terms using default parameters. The GO terms of each PHD-finger protein were classified into three main categories: biological process, molecular function, and cellular component. We selected the GO results at level 2 and visualized them using ggplot2 [45] in the R software.

### Phylogenetic analysis of PHD-finger genes

For phylogenetic analysis, multiple sequence alignment was performed with the re-annotated PHD-finger protein sequences using MAFFT v7.470 [46]. The alignments were trimmed by trimAL v1.4 (-gappyout) [47] to delete poorly aligned sequence regions. The phylogenetic tree was constructed from alignments, excluding any sequences containing only gaps, using the maximum-likelihood method with 1000 ultrafast bootstrap replicates in IQ-TREE v2.0.6 [48]. The tree was mid-point rooted and visualized using Interactive Tree of Life (iToL) v5 (http://itol.embl.de). Based on the tree, the PHD-finger proteins were clustered and divided into 14 subgroups (G1 ~ G14).

### Gene duplication analysis

To estimate the duplication time of PHD-finger genes, we identified recently duplicated PHD-finger gene pairs using DupGen_Finder [49]. The coding sequences of each gene pair were aligned using PRANK (-codon) [50]. To estimate duplication times of PHD-finger genes, synonymous substitution rates (Ks) were calculated using KaKs_Calculator 2.0 (-m MYN) [51].

### Chromosomal location and microsynteny analysis of PHD-finger genes

Chromosomal location of PHD-finger genes was obtained using GFF files from the re-annotation results of TGFam-Finder v1.20 [8] and visualized using MapChart [52]. With the exception of PHD-finger genes in the nongroup, the re-annotated genes were marked with the same subgroup colors in the phylogenetic tree.

Microsynteny analysis was conducted with genes in G6 located on chromosome 1 of pepper, potato, and tomato. All-by-all comparison for these genes was performed using BLASTP [53] to identify putative orthologous gene pairs. The genomic positions of syntenic genes were visualized using ChromoMap v0.2 [54] in the R software.

### Quantitative real-time PCR (qRT-PCR) analysis

We conducted qRT-PCR to validate the expression of newly identified PHD-finger genes using cDNA isolated from abiotic-stressed pepper leaves [55]. Primers (Table S4) were designed with the Primer3Plus online web tool (https://www.bioinformatics.nl/cgi-bin/primer3plus/primer3plus.cgi). The pepper ubiquitin gene (UBI-3) was used as a reference gene [56]. We selected six novel PHD-finger genes from pepper based on their high expression levels under abiotic stresses. qRT-PCR was carried out on a Mic qPCR Cycler (Bio Molecular System, Australia) using TB Green Premix Ex Taq II (Takara, Japan) with three technical replicates. PCR conditions were set as follows: 95 °C for 30 s for activation followed by 40 cycles of 95 °C for 5 s and 60 °C for 30 s. The relative expression values were calculated and normalized using the 2^−ΔΔCt^ method [57]. The bar plots in Fig. 5 were visualized with ggplot2 [45] in the R software.

### Expression analyses of pepper PHD-finger genes under abiotic stress

To analyze the expression of pepper PHD-finger genes under abiotic stress, we first downloaded previously reported RNA-Seq data from pepper leaves treated with various stresses [55]. These data contained results from four types of abiotic treatments (cold, heat, salt, and mannitol) at different time points (3, 6, 12, 24, and 72 h) with three biological replicates. Raw data were trimmed with CLC Assembly Cell (CLC Bio, Aarhus, Denmark) to filter out low-quality reads. The cleaned RNA-Seq data were mapped to the pepper genome using HISAT2 [58] (-dta -x). Expression levels of whole genes with newly identified PHD-finger genes in pepper were quantified and FPKM (Fragment Per Kilobase of transcript per Million mapped reads) values were calculated using StringTie [59] (-e -B -G). The overall expression profiles of the pepper PHD-finger genes under the various abiotic stresses were visualized with log2(FPKM + 1) values using pheatmap v1.0.12 (https://cran.r-project.org/web/packages/pheatmap/index.html) in the R software. We then identified DEGs with a *p*-value < 0.05 using Ballgown [60] from log_2_-transformed fold-change values that were calculated from averaged FPKM values.

To further investigate the expression pattern of pepper PHD-finger genes, we conducted clustering analysis with the DEGs using Mfuzz [61] in the R software. The number of clusters was set to four based on the k-means algorithm. Then, GO annotation of pepper DEGs in each cluster was performed using Omicsbox (version 1.4, https://www.biobam.com/omicsbox/). Enrichment test of GO terms in each cluster was performed using Fisher’s exact test (false discovery rates corrected *p*-value ≤ 0.01).

## Supplementary Information

**Additional file 1: Supplementary Figures. Figure S1.** Chromosomal locations of PHD-finger genes in the five genomes. (A-E) Gene names are listed next to each chromosome bar and written in the same colors of matched subgroups in phylogenetic tree. The PHD-finger genes in (A) *Arabidopsis* (230), (B) rice (191), (C) pepper (84), (D) potato (192), and (E) tomato (87) are mapped to chromosomes, respectively. **Figure S2.** Expression profiles of PHD-finger genes under various abiotic stresses. Normalized expression values (log2(FPKM +1)) are shown as a heat map. The colored scale bars in the upper right side of the heat map represents normalized expression values: red indicates high level of expression and green indicates low level of expression. Gene names are matched with subgroup colors in phylogenetic tree.

**Additional file 2: Supplementary Tables. Table S1.** List of the five plant genomic resources. **Table S2.** Detailed information on the re-annotated PHD-finger genes. **Table S3.** Description of integrated domain. **Table S4.** List of primers used in qRT-PCR.

## Data Availability

All data generated or analysed during this study are included in this published article and its supplementary information files.

## Abbreviations

PHD: Plant homeodomain
ID: Integrated domain
GO: Gene ontology
qRT-PCR: Quantitative real-time PCR
FPKM: Fragment Per Kilobase of transcript per Million mapped reads
DEG: Differentially expressed gene
FDR: False discovery rate
