# Supplementary material for: Identification of novel PHD-finger genes in pepper by genomic re-annotation and comparative analyses
Source: BMC Plant Biol. 2022 Apr 20;22:206. doi: 10.1186/s12870-022-03580-2 (PMC9020097; doi:10.1186/s12870-022-03580-2)
Supplement: Supplementary file 1 — Additional file 1: Supplementary Figures. Figure S1. Chromosomal locations of PHD-finger genes in the five genomes. (A-E) Gene names are listed next to each chromosome bar and written in the same colors of matched subgroups in phylogenetic tree. The PHD-finger genes in (A) Arabidopsis (230), (B) rice (191), (C) pepper (84), (D) potato (192), and (E) tomato (87) are mapped to chromosomes, respectively. Figure S2. Expression profiles of PHD-finger genes under various abiotic stresses. Normalized expression values (log2(FPKM +1)) are shown as a heat map. The colored scale bars in the upper right side of the heat map represents normalized expression values: red indicates high level of expression and green indicates low level of expression. Gene names are matched with subgroup colors in phylogenetic tree. [file 12870_2022_3580_MOESM1_ESM.pdf]

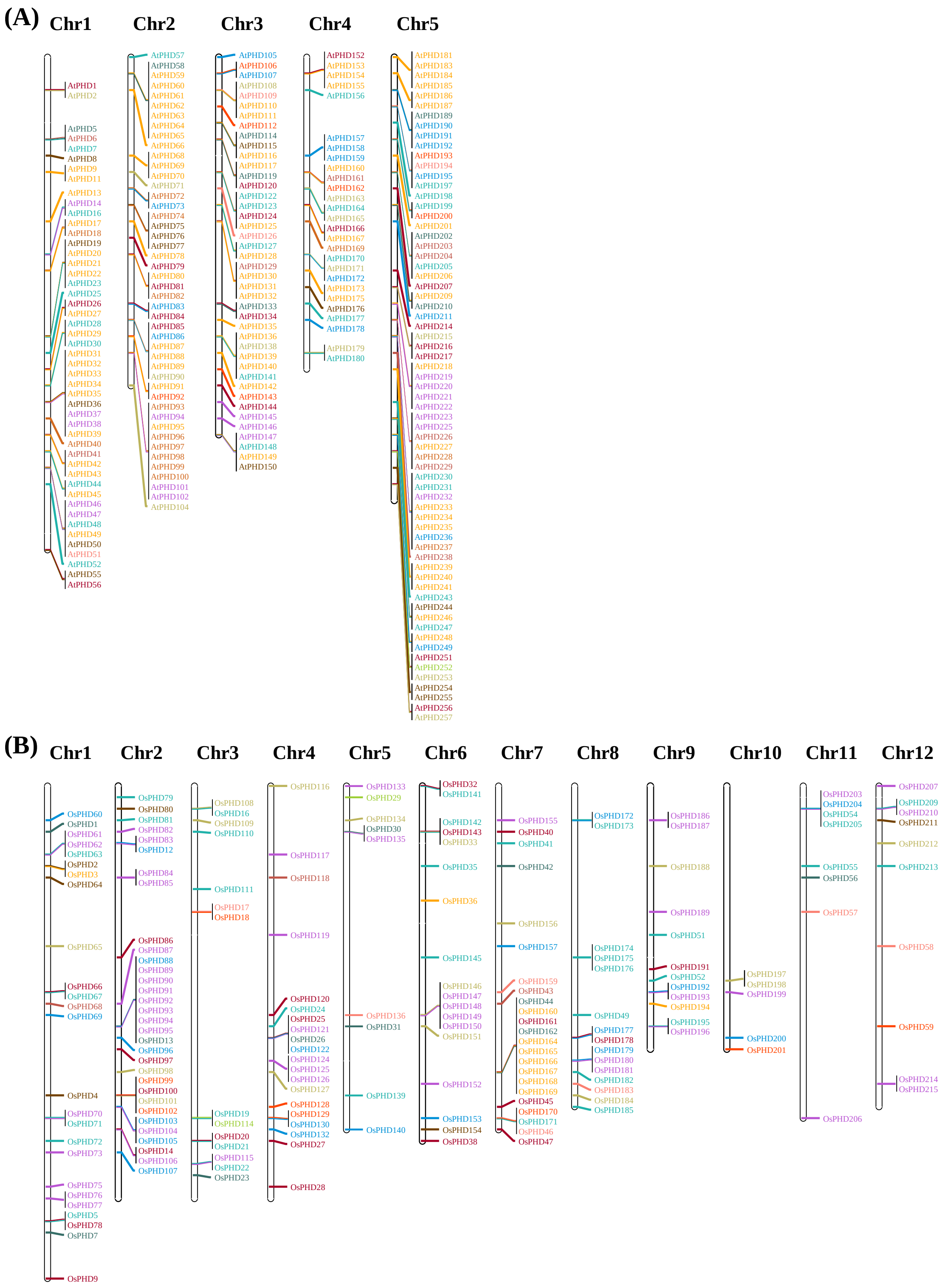

(A)
Chr1
AtPHD1
AtPHD2
AtPHD5
AtPHD6
AtPHD7
AtPHD8
AtPHD9
AtPHD11
AtPHD13
AtPHD14
AtPHD16
AtPHD17
AtPHD18
AtPHD19
AtPHD20
AtPHD21
AtPHD22
AtPHD23
AtPHD25
AtPHD26
AtPHD27
AtPHD28
AtPHD29
AtPHD30
AtPHD31
AtPHD32
AtPHD33
AtPHD34
AtPHD35
AtPHD36
AtPHD37
AtPHD38
AtPHD39
AtPHD40
AtPHD41
AtPHD42
AtPHD43
AtPHD44
AtPHD45
AtPHD46
AtPHD47
AtPHD48
AtPHD49
AtPHD50
AtPHD51
AtPHD52
AtPHD55
AtPHD56
Chr2
AtPHD57
AtPHD58
AtPHD59
AtPHD60
AtPHD61
AtPHD62
AtPHD63
AtPHD64
AtPHD65
AtPHD66
AtPHD68
AtPHD69
AtPHD70
AtPHD71
AtPHD72
AtPHD73
AtPHD74
AtPHD75
AtPHD76
AtPHD77
AtPHD78
AtPHD79
AtPHD80
AtPHD81
AtPHD82
AtPHD83
AtPHD84
AtPHD85
AtPHD86
AtPHD87
AtPHD88
AtPHD89
AtPHD90
AtPHD91
AtPHD92
AtPHD93
AtPHD94
AtPHD95
AtPHD96
AtPHD97
AtPHD98
AtPHD99
AtPHD100
AtPHD101
AtPHD102
AtPHD104
Chr3
AtPHD105
AtPHD106
AtPHD107
AtPHD108
AtPHD109
AtPHD110
AtPHD111
AtPHD112
AtPHD114
AtPHD115
AtPHD116
AtPHD117
AtPHD119
AtPHD120
AtPHD122
AtPHD123
AtPHD124
AtPHD125
AtPHD126
AtPHD127
AtPHD128
AtPHD129
AtPHD130
AtPHD131
AtPHD132
AtPHD133
AtPHD134
AtPHD135
AtPHD136
AtPHD138
AtPHD139
AtPHD140
AtPHD141
AtPHD142
AtPHD143
AtPHD144
AtPHD145
AtPHD146
AtPHD147
AtPHD148
AtPHD149
AtPHD150
Chr4
AtPHD152
AtPHD153
AtPHD154
AtPHD155
AtPHD156
AtPHD157
AtPHD158
AtPHD159
AtPHD160
AtPHD161
AtPHD162
AtPHD163
AtPHD164
AtPHD165
AtPHD166
AtPHD167
AtPHD169
AtPHD170
AtPHD171
AtPHD172
AtPHD173
AtPHD175
AtPHD176
AtPHD177
AtPHD178
AtPHD179
AtPHD180
Chr5
AtPHD181
AtPHD183
AtPHD184
AtPHD185
AtPHD186
AtPHD187
AtPHD189
AtPHD190
AtPHD191
AtPHD192
AtPHD193
AtPHD194
AtPHD195
AtPHD197
AtPHD198
AtPHD199
AtPHD200
AtPHD201
AtPHD202
AtPHD203
AtPHD204
AtPHD205
AtPHD206
AtPHD207
AtPHD209
AtPHD210
AtPHD211
AtPHD214
AtPHD215
AtPHD216
AtPHD217
AtPHD218
AtPHD219
AtPHD220
AtPHD221
AtPHD222
AtPHD223
AtPHD225
AtPHD226
AtPHD227
AtPHD228
AtPHD229
AtPHD230
AtPHD231
AtPHD232
AtPHD233
AtPHD234
AtPHD235
AtPHD236
AtPHD237
AtPHD238
AtPHD239
AtPHD240
AtPHD241
AtPHD243
AtPHD244
AtPHD246
AtPHD247
AtPHD248
AtPHD249
AtPHD251
AtPHD252
AtPHD253
AtPHD254
AtPHD255
AtPHD256
AtPHD257
(B)
Chr1
OsPHD60
OsPHD1
OsPHD61
OsPHD62
OsPHD63
OsPHD2
OsPHD3
OsPHD64
OsPHD65
OsPHD66
OsPHD67
OsPHD68
OsPHD69
OsPHD4
OsPHD70
OsPHD71
OsPHD72
OsPHD73
OsPHD75
OsPHD76
OsPHD77
OsPHD5
OsPHD78
OsPHD7
OsPHD9
Chr2
OsPHD79
OsPHD80
OsPHD81
OsPHD82
OsPHD83
OsPHD12
OsPHD84
OsPHD85
OsPHD86
OsPHD87
OsPHD88
OsPHD89
OsPHD90
OsPHD91
OsPHD92
OsPHD93
OsPHD94
OsPHD95
OsPHD13
OsPHD96
OsPHD97
OsPHD98
OsPHD99
OsPHD100
OsPHD101
OsPHD102
OsPHD103
OsPHD104
OsPHD105
OsPHD14
OsPHD106
OsPHD107
Chr3
OsPHD108
OsPHD16
OsPHD109
OsPHD110
OsPHD111
OsPHD17
OsPHD18
OsPHD19
OsPHD114
OsPHD20
OsPHD21
OsPHD115
OsPHD22
OsPHD23
Chr4
OsPHD116
OsPHD117
OsPHD118
OsPHD119
OsPHD120
OsPHD24
OsPHD25
OsPHD121
OsPHD26
OsPHD122
OsPHD124
OsPHD125
OsPHD126
OsPHD127
OsPHD128
OsPHD129
OsPHD130
OsPHD132
OsPHD27
OsPHD28
Chr5
OsPHD133
OsPHD29
OsPHD134
OsPHD30
OsPHD135
OsPHD136
OsPHD31
OsPHD139
OsPHD140
Chr6
OsPHD32
OsPHD141
OsPHD142
OsPHD143
OsPHD33
OsPHD35
OsPHD36
OsPHD145
OsPHD146
OsPHD147
OsPHD148
OsPHD149
OsPHD150
OsPHD151
OsPHD152
OsPHD153
OsPHD154
OsPHD38
Chr7
OsPHD155
OsPHD40
OsPHD41
OsPHD42
OsPHD156
OsPHD157
OsPHD159
OsPHD43
OsPHD44
OsPHD160
OsPHD161
OsPHD162
OsPHD164
OsPHD165
OsPHD166
OsPHD167
OsPHD168
OsPHD169
OsPHD45
OsPHD170
OsPHD171
OsPHD46
OsPHD47
Chr8
OsPHD172
OsPHD173
OsPHD174
OsPHD175
OsPHD176
OsPHD49
OsPHD177
OsPHD178
OsPHD179
OsPHD180
OsPHD181
OsPHD182
OsPHD183
OsPHD184
OsPHD185
Chr9
OsPHD186
OsPHD187
OsPHD188
OsPHD189
OsPHD51
OsPHD191
OsPHD52
OsPHD192
OsPHD193
OsPHD194
OsPHD195
OsPHD196
Chr10
OsPHD197
OsPHD198
OsPHD199
OsPHD200
OsPHD201
Chr11
OsPHD203
OsPHD204
OsPHD54
OsPHD205
OsPHD55
OsPHD56
OsPHD57
OsPHD206
Chr12
OsPHD207
OsPHD209
OsPHD210
OsPHD211
OsPHD212
OsPHD213
OsPHD58
OsPHD59
OsPHD214
OsPHD215

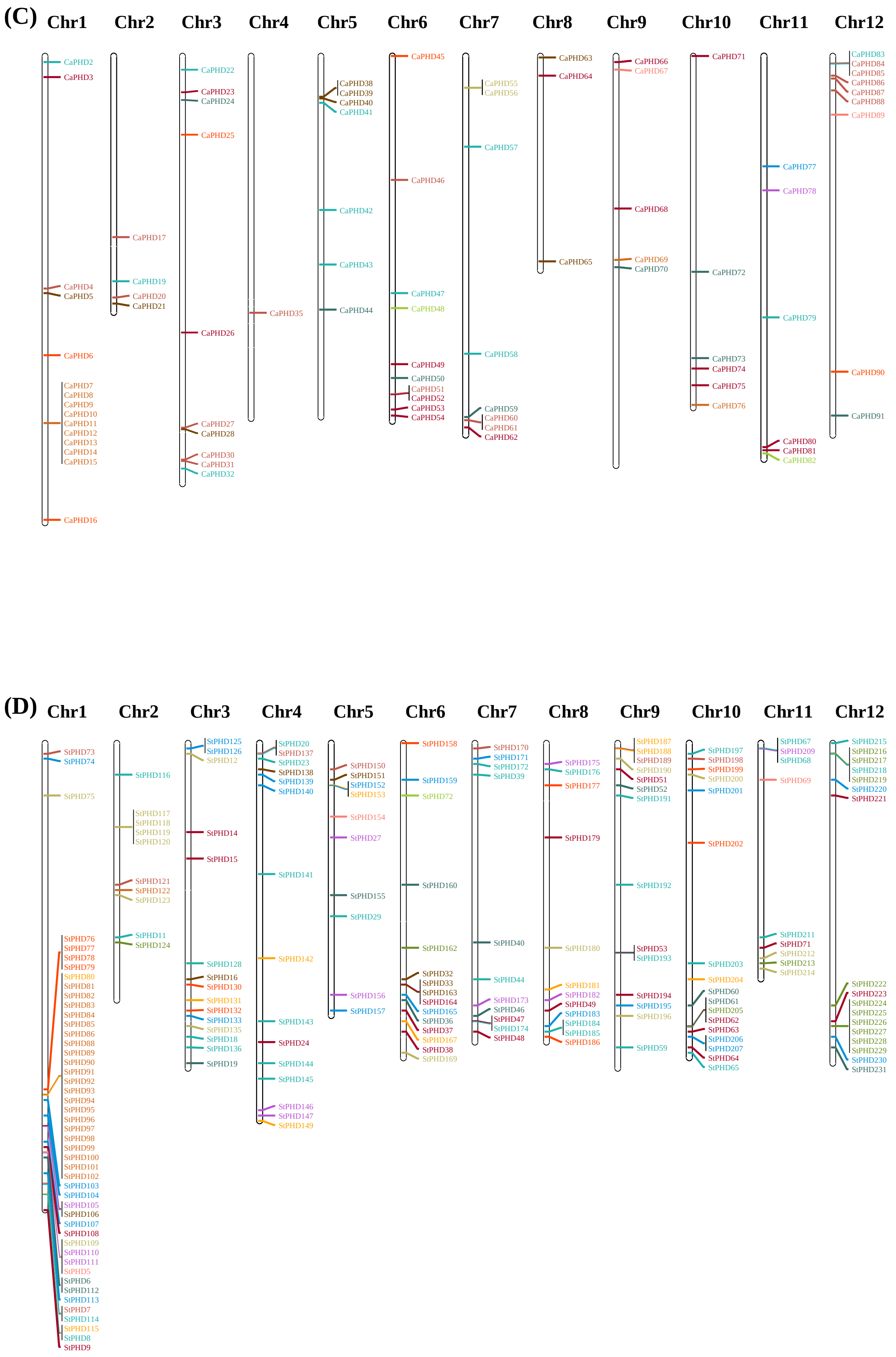

(C)
Chr1
Chr2
Chr3
Chr4
Chr5
Chr6
Chr7
Chr8
Chr9
Chr10
Chr11
Chr12
CaPHD2
CaPHD3
CaPHD4
CaPHD5
CaPHD6
CaPHD7
CaPHD8
CaPHD9
CaPHD10
CaPHD11
CaPHD12
CaPHD13
CaPHD14
CaPHD15
CaPHD16
CaPHD17
CaPHD19
CaPHD20
CaPHD21
CaPHD22
CaPHD23
CaPHD24
CaPHD25
CaPHD26
CaPHD27
CaPHD28
CaPHD30
CaPHD31
CaPHD32
CaPHD35
CaPHD38
CaPHD39
CaPHD40
CaPHD41
CaPHD42
CaPHD43
CaPHD44
CaPHD45
CaPHD46
CaPHD47
CaPHD48
CaPHD49
CaPHD50
CaPHD51
CaPHD52
CaPHD53
CaPHD54
CaPHD55
CaPHD56
CaPHD57
CaPHD58
CaPHD59
CaPHD60
CaPHD61
CaPHD62
CaPHD63
CaPHD64
CaPHD65
CaPHD66
CaPHD67
CaPHD68
CaPHD69
CaPHD70
CaPHD71
CaPHD72
CaPHD73
CaPHD74
CaPHD75
CaPHD76
CaPHD77
CaPHD78
CaPHD79
CaPHD80
CaPHD81
CaPHD82
CaPHD83
CaPHD84
CaPHD85
CaPHD86
CaPHD87
CaPHD88
CaPHD89
CaPHD90
CaPHD91
(D)
Chr1
Chr2
Chr3
Chr4
Chr5
Chr6
Chr7
Chr8
Chr9
Chr10
Chr11
Chr12
StPHD73
StPHD74
StPHD75
StPHD76
StPHD77
StPHD78
StPHD79
StPHD80
StPHD81
StPHD82
StPHD83
StPHD84
StPHD85
StPHD86
StPHD88
StPHD89
StPHD90
StPHD91
StPHD92
StPHD93
StPHD94
StPHD95
StPHD96
StPHD97
StPHD98
StPHD99
StPHD100
StPHD101
StPHD102
StPHD103
StPHD104
StPHD105
StPHD106
StPHD107
StPHD108
StPHD109
StPHD110
StPHD111
StPHD5
StPHD6
StPHD112
StPHD113
StPHD7
StPHD114
StPHD115
StPHD8
StPHD9
StPHD116
StPHD117
StPHD118
StPHD119
StPHD120
StPHD121
StPHD122
StPHD123
StPHD11
StPHD124
StPHD125
StPHD126
StPHD12
StPHD14
StPHD15
StPHD128
StPHD16
StPHD130
StPHD131
StPHD132
StPHD133
StPHD135
StPHD18
StPHD136
StPHD19
StPHD20
StPHD137
StPHD23
StPHD138
StPHD139
StPHD140
StPHD141
StPHD142
StPHD143
StPHD24
StPHD144
StPHD145
StPHD146
StPHD147
StPHD149
StPHD150
StPHD151
StPHD152
StPHD153
StPHD154
StPHD27
StPHD155
StPHD29
StPHD156
StPHD157
StPHD158
StPHD159
StPHD72
StPHD160
StPHD162
StPHD32
StPHD33
StPHD163
StPHD164
StPHD165
StPHD36
StPHD37
StPHD167
StPHD38
StPHD169
StPHD170
StPHD171
StPHD172
StPHD39
StPHD40
StPHD44
StPHD173
StPHD46
StPHD47
StPHD174
StPHD48
StPHD175
StPHD176
StPHD177
StPHD179
StPHD180
StPHD181
StPHD182
StPHD49
StPHD183
StPHD184
StPHD185
StPHD186
StPHD187
StPHD188
StPHD189
StPHD190
StPHD51
StPHD52
StPHD191
StPHD192
StPHD53
StPHD193
StPHD194
StPHD195
StPHD196
StPHD59
StPHD197
StPHD198
StPHD199
StPHD200
StPHD201
StPHD202
StPHD203
StPHD204
StPHD60
StPHD61
StPHD205
StPHD62
StPHD63
StPHD206
StPHD207
StPHD64
StPHD65
StPHD67
StPHD209
StPHD68
StPHD69
StPHD211
StPHD71
StPHD212
StPHD213
StPHD214
StPHD215
StPHD216
StPHD217
StPHD218
StPHD219
StPHD220
StPHD221
StPHD222
StPHD223
StPHD224
StPHD225
StPHD226
StPHD227
StPHD228
StPHD229
StPHD230
StPHD231

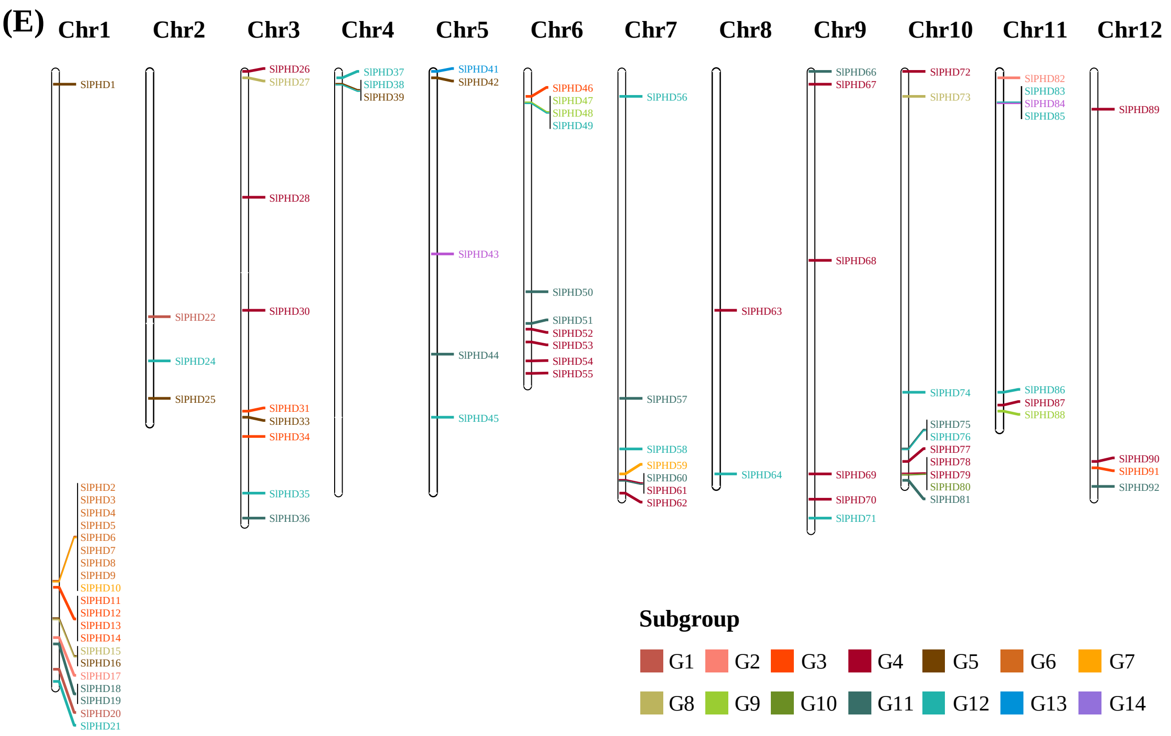

Figure S1. Chromosomal locations of PHD-finger genes in the five genomes. (A-E) Gene names are listed next to each chromosome bar and written in the same colors of matched subgroups in phylogenetic tree. The PHD-finger genes in (A) *Arabidopsis* (230), (B) rice (191), (C) pepper (84), (D) potato (192), and (E) tomato (87) are mapped to chromosomes, respectively.

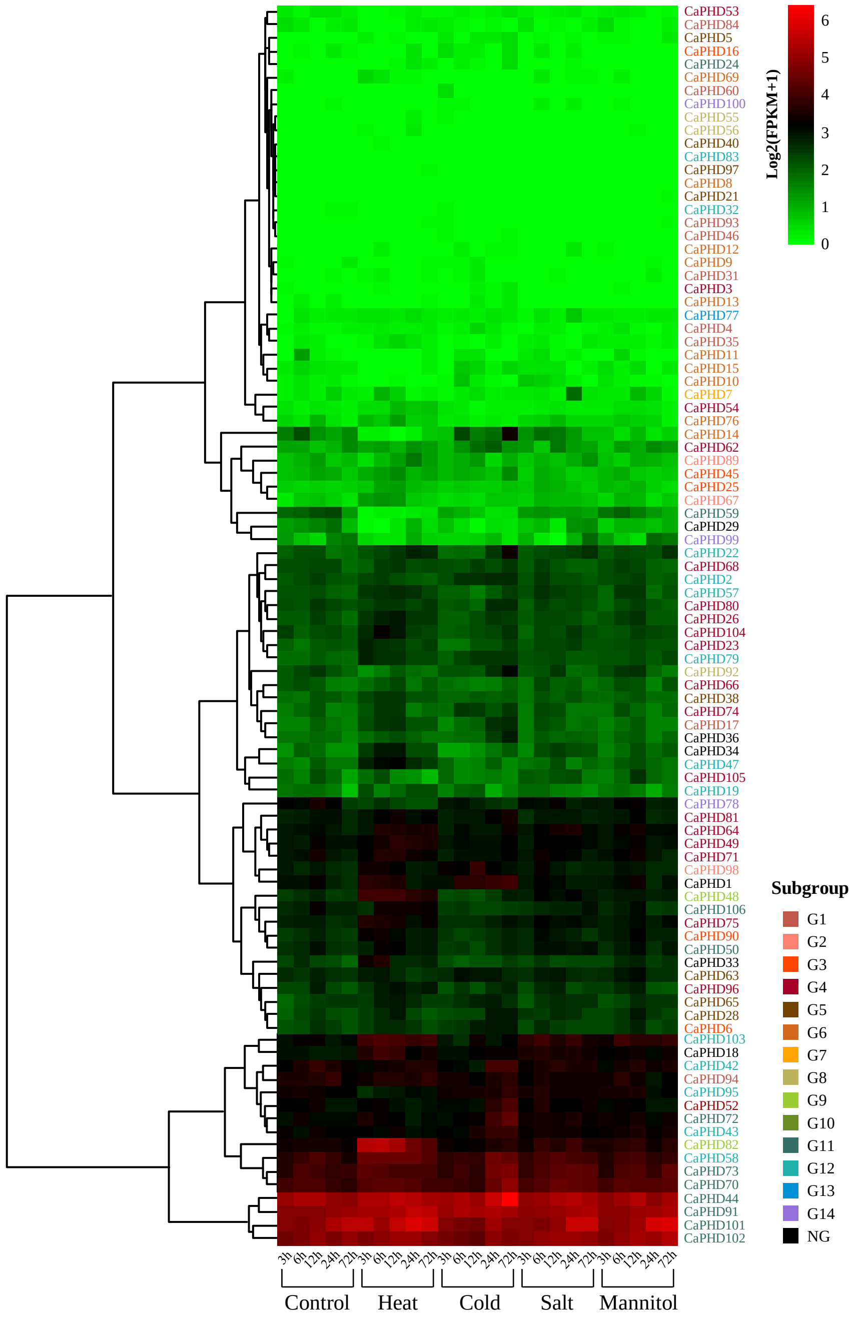

Figure S2. Expression profiles of PHD-finger genes under various abiotic stresses. Normalized expression values (log2(FPKM +1)) are shown as a heat map. The colored scale bars in the upper right side of the heat map represents normalized expression values: red indicates high level of expression and green indicates low level of expression. Gene names are matched with subgroup colors in phylogenetic tree.
